# Initial Real Time Coping by African American Christians During the Coronavirus Pandemic (COVID-19)

**DOI:** 10.1177/15423050211027523

**Published:** 2021-06-29

**Authors:** Lee N. June, Shirley A. June

**Affiliations:** Department of Psychology, Honors College, Michigan State University, East Lansing, MI, USA; Retired, Michigan State University, East Lansing, MI, USA

**Keywords:** religious coping, spiritual coping, African American spirituality, COVID-19 pandemic

## Abstract

Seventy-three telephone interviews during the initial COVID-19 shutdown explored coping strategies among African American Christians. Open-ended questions and Likert-type scale items assessed faith, advice given, helpful Scriptures, worship practices, belief in God, and religion and spirituality’s importance. Most participants reported coping well and following governmental guidelines. Frequent church attendance before the shutdown was significantly associated with less worrying and being less anxious. Married individuals also reported less worrying. We discuss limitations, future research, and implications.

This study explored how African American Christians were coping with the coronavirus pandemic (COVID-19) between April 17 and April 28, 2020. The overall goal was to capture in real time participants’ experiences with COVID-19 and present implications for counseling, ministry, and pastoral care. The study was conducted during the governmental shut down and as stay-at-home orders were in effect.

Media reports and polling data are readily available regarding the general impact of COVID-19 (Pew Forum, 2020a, 2020b; The Associated Press-NORC Center for Public Affairs Research, 2020a, 2020b; *USA Today*, 2020). Impacts include death of family members and other loved ones, disruptions in personal and family lives, business closures, and major adjustments in modes of operations of individuals and churches. These reports and data are helpful, but do not provide the needed in-depth information regarding coping methods and the specific impacts experienced by individuals in the religious community. Counselors, pastoral care professionals, as well as other mental health practitioners could greatly profit from knowing more about the impact of this crisis and how they might better serve their constituents. Additionally, such a study of religious coping is extremely important because a large percentage of the American population indicates that religion and spirituality are important in their lives (Pew Forum, 2014).

Religion and spirituality operate within and outside of formal religious settings and provide numerous and unique resources that aid individuals in dealing with a variety of life issues, including crises. Before COVID-19, many Americans received spiritual guidance mainly through face-to-face interaction. With the COVID-19 pandemic, this practice is less available. Studies conducted during crises and traumas have found that individuals experience various stressors (Poulin et al., 2009). Determining unique stressors during COVID-19, however, warrants its own exploration.

Gall and Guirguis-Younger (2013) literature review of religious and spiritual coping noted that highly religious and spiritual individuals depended on religion and spirituality as coping strategies in times of stress. These strategies were linked to better psychological and physical health. Others make similar observations about coping and buffering effects of religion and spirituality (Hood et al., 2018; Koenig, 2012; Molock et al., 2006; Pan et al., 2012; Soenke et al., 2013). Mattis (2002, 2013) and Taylor et al. (2004) document unique coping and buffering effects of religion and spirituality in the lives of African Americans. Others have addressed the broader historical importance of the church (DuBois, 1899; June, 2008; Lincoln, 1974; Lincoln & Mamiya, 1990; Mays & Nicholson, 1933; Woodson, 1921). There is consensus that the church plays a unique role for African Americans.

Further, polling data during the coronavirus pandemic show a differential impact of religion and spirituality in the lives of African Americans in that they report at a higher rate than other racial/ethnic groups, that their faith increased during the pandemic (Gecewicg, 2020). Additionally, Pew Forum (2009) reported that African Americans indicate a higher importance of religion in their lives (79%) than does the general population (56%). African Americans attend religious services at least once weekly at a higher rate (53% versus 39%); and the frequency of daily prayer was higher (76% versus 58%). Barna (2009, 2011) reported similar differences.

Given the unique role of religion and spirituality within the African American community, this study specifically gives voice to and explores changes and adaptations made during COVID-19. How are individuals in the African American community coping? What role is faith playing? What advice is considered important to give to others? How are worship practices being maintained? What adaptations are occurring regarding prayer, Bible reading, and meditation? What lessons are being learned in the absence of regular in-person religious service attendance? To what extent are individuals worrying and anxious? Finding answers to these questions will provide important information for effective counseling, ministry, and pastoral care during crises.

## Method

### Survey Procedure

Approved by the University’s Institutional Review Board, the study was limited to telephone or other electronic participation due to COVID-19 restrictions in place for person-to- person research contact. Participants were identified through snowballing and convenience sampling. The senior author read a Research Participation and Informed Consent Form to participants, and upon voluntary agreement, conducted and hand recorded telephone interviews that lasted up to 20 minutes. When necessary, the interview was paused to fully record the responses, or responses were read back to participants to assure accuracy.

### Participants

Seventy-three individuals who self-identified as African Americans and Christians were included in the study. Forty of the participants (54.8%) were from Michigan. The other states included Alabama, California, Florida, Illinois, Indiana. Maryland, New Jersey, New York, North Carolina, Ohio, South Carolina, Tennessee, and Texas. By region, 50 participants were from the Midwest, 18 from the South, four from the East, and one from the West. Numerous occupations were reported, and 25 participants were retired. Participants described their religious affiliations with labels indicating a Christian orientation. Three racial/ethnic descriptors were used: African American (n = 45, 61.6%), Black or African American (n = 14, 19.2%), and Black (n = 14, 19.2%). Since most of the sample self-described as African American, this descriptor is used throughout this article. Table 1 shows other characteristics of the sample.

**Table 1.** Sample characteristics.

Variable	N	Percent
Sex		
Male	38	52.1
Female	35	47.9
Age (year born)		
Before 1944	12	16.4
1944–1964	26	35.6
1965–1979	11	15.1
1980–1994	11	15.1
1995–2015	13	17.8
Marital status		
Single	31	42.5
Married	31	42.5
Divorced	8	11.0
Widow/widower	3	4.1
Other	0	0
Church attendance		
Weekly	51	69.9
Twice monthly	7	9.6
Monthly	7	9.6
Other	8	11.0

### The Measures

Six opened-ended questions were used along with a demographic questionnaire.

Additionally, eight Likert-type scale items were developed specifically for this study. Respondents indicated whether they *strongly agreed*, *agreed*, *disagreed*, or *strongly disagreed* with each item. All items are presented in Table 2.

**Table 2.** Quantitative, qualitative, and demographic measures.

Measures	Items
Likert-type scale items	I believe in God or a Divine Being.
	I believe there is a God or Divine Being.
	I would describe myself as a religious person.
	I would describe myself as a spiritual person.
	Religion is important to me.
	Spirituality is important to me.
	During the coronavirus pandemic, I would describe myself as anxious.
	During the coronavirus pandemic, I would describe myself as worrying.
Qualitative items	Describe how you are coping during the coronavirus pandemic.
	How has your religious faith helped you cope during this coronavirus pandemic?
	What scriptural passages, if any, are you finding helpful during this coronavirus pandemic?
	What advice would you give to Christian/believers during this coronavirus pandemic?
	How are you maintaining your worship practices during this period?
	Have any of these practices changed during this period, and if so, how? • Prayer • Bible reading • Personal meditation
Demographic survey	During normal times, how often do you attend church services? (circle one) • Weekly • Twice monthly • Monthly • Other (please specify)
	Your race/ethnicity
	Your sex
	Your religious affiliation
	Your occupation
	Your state of residence
	Your marital status • Single • Married • Divorced • Widow/widower • Other (please specify)
	Please indicate the year in which you were born (circle one). • Before 1944 • 1944–1964 • 1965–1979 • 1980–1994 • 1995–2015

## Results

### Qualitative Responses and Analyses

Qualitative analyses were assisted by use of NVivo. Verbatim responses to open-ended questions reveal participants’ reports regarding coping in general, the role of faith in coping, which scriptural passages were helpful during the pandemic, advice they would give others, how worship practices were being maintained, and whether certain worship practices had changed.

We present and comment on representative verbatim responses to these questions.

#### Question One – “How Are You Coping With the Coronavirus Pandemic?”

Most respondents reported that they were coping well and adhering to governmental guidelines (for example, wearing masks, and social distancing). They indicated limiting normal activities; they were staying home, except for necessary grocery shopping, medicine outings, and taking occasional walks. Some indicated difficulty initially adjusting before settling into their routines. Many relished this time as an opportunity to pause and be still. For those coping well, typical responses were:

On average well (#2); Coping well. Trying to get used to staying at home (#11); I use the lockdown time to pray and count my blessings (#18); Doing well (#26); I am well. My anxiety has lessened right now (#42); Actually, I have been coping very well. I take precautions (#45); I am coping well. Well, I am a child of God and I do not have to worry about those things. Of course, I am being careful using the gloves and mask (#46); and Coping really well (#54).

Respondents reported engaging in a variety of activities that assisted them in coping well.

Representative responses were:

By praying, reading my Bible … My booklet … talking to friends (#5); I am coping by studying … keeping in contact with my friends … texting and calling each other … watching movies … reading a bit … doing a Bible study with two friends … working out more consistently (#10); … I also use the time to work in my yard, plant and I enjoy it (#18); Doing service and dial-up (#19); By exercising mentally, by reading (#30); I meditate, I listen to soothing music. I go out every day and take a long walk, weather permitting (#35); … doing a lot of zoom meetings and conference calls, Bible study … 

(#37); I am watching movies, playing bridge online, also Bid Whist … also attending Bible study and church services online (#39); By keeping busy. I try to schedule some activity to do at home every day. I read scriptures and contact friends, family, and church members (#43); I still workout … take daily walks outside or on my treadmill (#45); Eating (#48); … I will take a trip to the park, sometimes just sit there. Other times to walk and stretch” (#49); I have a routine self-care in place … to address mind, body and spirit. For mind, I have been engaging a lot of articles about resiliency … for spiritual practices, I do morning and evening prayer meditation … for body, exercising and eating well (#52); I have just grown to be in the stillness provided in these things (#58); I am coping by running, every day, reading, and playing the piano, preparing to graduate, and hanging with family (#62); … I use the time to hone in and spend time with my wife and kids. I am using the time to spend time and enjoy in every way (#63); I have been on Amazon and got free copies of books to read … helping my father … Every morning I pray the promise- Psalm 91 over my family and for people that have the COVID-19 that they may recover (#66); I am taking time to enjoy it while I can. I am primarily doing that by watching Netflix as well (68); and I am spending my time consoling people who are going through rough times (#73).

While most individuals reported that they were coping well, typical responses for those not coping well were:

Still adjusting because each week there is something different (#4); Fairly ok. I am not functioning on all cylinders/maximum capacity because I cannot go out being in … in a condo … (#13); This is a lifestyle change for me … this feels like house arrest to me. I am reflecting on the spiritual meaning of this (#41); and I do not have any healthy coping mechanisms right now. Still trying to figure that out … (#57).

#### Open-Ended Question Two – “How Has Your Religious Faith Helped You to Cope During the Coronavirus Pandemic?”

Responses to this question suggest strong buffering and protective effects of faith as evidenced by words such as “control,” “security,” “comfort,” and “inner peace.”

That is the only way I am getting through (#1); That’s my comfort (#4); I could not make it through if it had not been for my faith and trust in the Lord (#5); Very much so. Even though I do not fully understand, I know my faith has allowed me to understand that God is in control of all things. Even though I don’t understand, I trust in the fact that He does. That brings peace (#6); It helps a lot. It helps me to have an inner peace despite all the chaos with the news and people getting sick … (#10); If I did not know Jesus Christ as my savior and Lord, I would have probably lost my mind (#15); Oh, without my faith, I would have been a basket case (#28); My faith is my primary resource … (#41); My faith has played a tremendous role in helping me to cope. Because it has provided me an extra blanket of security outside of my parents (#62); and about 3000% … (#73).

#### Open-Ended Question Three – “What Scripture Passages, If Any, Are You Finding Helpful During This Coronavirus Pandemic?”

Psalms was the most frequently referenced book of the Bible. Of the Psalms, Psalm 91 was the most frequent at 12, while Psalm 23 was mentioned six times. Other Psalms mentioned more than once were Psalms 16, 34, 46, and 121. Regarding Philippians, the most referenced verses were all or part of Philippians 4:6–9. For Matthew, the most frequently mentioned passage was 6:33; for Joshua, it was 1:9; for Proverbs it was 3:5–6; and for 2 Chronicles, it was 7:14. The frequencies for all books mentioned four or more times were Psalms (n = 48), Philippians (n = 9), Isaiah (n = 8), Matthew (n = 8), Joshua (n = 5), Proverbs (n = 5), and 2 Chronicles (n = 4).

*Open-Ended Question Four – “What Advice Would You Give to Christians/Believers During the Coronavirus Pandemic?”*

The advice most often given was reflected by the following phrases:

If you don’t know Jesus, find him right away … (#1); To stay connected to our Lord for Guidance. God is allowing this to happen for a reason and we should understand the reason and work with that (#2); Trust in the Lord. Listen to what he has to say to you

(#4); To trust in the Lord, to pray … (#5); As Christians, our main role is to follow what

the experts are telling us to do. Then to put our trust in God after that (#6); To continue to trust in God. Stay faithful and obedient (#8); Spend more time with God and his word and also listen to what he has to say instead of me talking to him predominantly (#65); To not lose faith. There is a God and that he will make a way out of no way (#68); To put their trust in the Lord and not in confidence of what others say. Put confidence in the

Lord, don’t do stupid things, don’t try and test the Lord by disobeying the doctors and scientists … God teaches us to obey the laws of the land. Don’t walk around in fear. It is using common sense. Some of the things preachers are saying don’t stand up. We need to listen to authorities and not hold services (#73).

In these responses, one can see the theme of stay connected/trust/and connect to God as the primary modes of advice.

#### Open-Ended Question Five – “How Are You Maintaining Your Worship Practices During This Period?”

The words Bible (n = 27) and online (n = 26) dominated the responses. Specific reference was made to Facebook, Zoom, Bible apps, and YouTube. Devotional materials were often utilized. Respondents also relied heavily on prayer (n = 19) and Bible study. Respondents relished having more time to study and reflect on the Bible.

#### Open-Ended Question Six – “Have Your Prayer Life, Bible Reading, and Personal Meditation Changed During This Period, and If So, How?”

This question had three parts. In terms of prayer life, respondents overwhelmingly said yes, indicating that their prayer life increased. Representative responses were:

I am focusing more on all the things that come from Biblical prophecy (#1); It is much deeper (#6); My prayer included others I didn’t before (politicians … ) (#5); It is more frequent and regular; Yes, slightly more. Praying in conjunction with the news (3); Prayer is more refined (#16); My prayer life has not changed, but my prayer subject has focused a lot on the pandemic (#23); Because I am sitting, I pray more (# 38); and My prayer life has become stronger(#51).

For changes in Bible reading, representative responses were:

Yes, it has. I am focusing more on all the things that come from Bible prophecy (#1);

Increased as a result of watching news and more services online (#2); Yes, I go to authors, I am doing a Bible study (#4); I now read day and night. I am very grateful (#15); Have more time to read (#20); Yes, I try to look to scripture for trials and tribulations God’s people went through similar to now (#24); Yes, I read more frequently because I am not at a work site (#25); I journal, so I am going through my journal and reviewing Scriptures recorded there that have given me direction in previous times of challenges (#41); and More intense Bible reading. I have more time to do it (#65).

Twenty-three respondents indicated no change or that Bible reading remained the same.

For meditation, typical responses beyond the word “yes” were:

Yes, I find myself meditating on Biblical prophecy (#1); Yes, it has increased. I spend more time talking to God and meditating (#8); Yes, it has increased. I do it with more focus (#12); Yes, I have more time to do that (#18, #36); Yes, that has really increased.

That’s the best way I handle my anxiety (#32); and Yes, I am maintaining my practice of getting up early in the morning and being silent before the Lord (#41).

Twenty-nine individuals indicated that their meditation practices did not change, have remained the same, or that they do not do personal meditations. Several individuals answered by simply saying “no” followed by brief explanations.

### Quantitative Analyses

Table 3 contains the means, medians, modes, standard deviations, variances, and ranges for Likert-scale type items that were responded to on a four-point scale- *strongly agree*, (4), *agree* (3), *disagree* (2), and *strongly disagree* (1). Since the items “I believe in God, or a divine being” and “I believe there is a God, or a divine being,” both had means of 3.99 (one person responding *agree* and all other responses to this item were *strongly agree*), further analyses used only the item “I believe in God or a divine being.” An independent samples *t*-test on the overall sample revealed no significant mean difference by sex for the Likert scale type items. Likewise, one-way ANOVAs for the overall sample showed no significant differences by overall church attendance, year born, and marital status for any of the Likert-type scale items. A significant level of *p* equal to or less than .05 (two-tailed) was set for all analyses.

**Table 3.** Likert-type scale items: Means, medians, modes, standard deviations, variances, and ranges.

	Believe in God	Believe there is a God	Spirituality Important	Religion Important	Am a Spiritual Person	Am a Religious person	Anxious	Worrying
*Mean*	3.99	3.99	3.78	3.28	3.75	3.18	2.25	2.14
*Median*	4.00	4.00	4.00	3.00	4.00	3.00	2.00	2.00
Mode	4	4	4	4	4	4	2	2
*SD*	.117	.117	.419	.773	.467	.861	.910	.855
Variance	.014	.014	.175	.598	.218	.742	.827	.731
Range	1	1	1	3	2	3	3	3
*N*	73	73	72	72	72	72	73	73
Missing	0	0	1	1	1	1	0	0

Significant positive Pearson correlations were between the items “spirituality is important to me” and “religion is important to me” (*r* = .324); “I am a spiritual person” and “spirituality is important to me” (*r* = .648); and “I am a religious person” and “spirituality is important to me” (*r* = .269). Additionally, there were significant positive correlations between “I am a spiritual person” and “religion is important to me” (*r* = .273); and “I am a religious person” and “religion is important to me” (*r* = .812). The correlation between worrying and anxious was *r* = .599. For anxious and worrying, because of small numbers in some of the attendance categories, two groups were created and compared (those who attended church services at least weekly during normal times and those who attended twice monthly or less) for anxious and worrying. There were significant negative correlations between worrying and church attendance combined (*r* = −.281) and between anxious and church attendance combined (*r* = −.250). All correlations for the Likert-type scale items and attendance combined are reported in Table 4.

**Table 4.** Correlations (Likert-type scales items and attendance combined).

	1	2	3	4	5	6	7
1. Belief in God	–						
2. Spirituality Imp	−.063	–					
3. Religion Imp	−.112	.324**	–				
4. Spiritual Person	−.064	.648**	.273*	–			
5. Religious Person	.025	.269*	.812**	.394**	–		
6. Anxious	−.098	.157	−.026	−.042	−.044	–	
7. Worrying	.019	.215	.106	.027	.077	.599**	–
8. Attendance Combined	−.077	.008	.083	.032	.034	−.250*	−.281*

*Correlation is significant at the 0.05 level (2-tailed).

**Correlation is significant at the 0.01 level (2-tailed).

Since there were significant negative correlations between both worrying and church attendance combined, and anxious and church attendance combined, independent samples *t-* tests were performed on these variables. The result for anxious was *t* = 2.179, *df* = 71, *p* = .033 (*M* = 2.10 weekly: *M* = 2.59 less than weekly). For worrying the result was *t* = 2.465, *df* = 71, *p* =. 016 (*M* = 1.98 weekly; *M* = 2.50 less than weekly). Thus, those who attended church services before the pandemic at the rate of once a week or more reported worrying significantly less and being significantly less anxious than those who attended twice monthly or less.

Given that the numbers were uneven for the marital status categories overall and there was an equal number of persons who were married or single, independent samples *t-* tests were conducted for attendance, anxious, and worrying for these two groups. There was a significant difference, with those married attending church services at more frequent rate than those single (*M* = .8387 versus .5161; *t =* 2.847; *df* = 60; *p* = .006). There was also a significant difference for those married versus single in terms of worrying (*M* = 2.39 versus 1.90; *t* = 2.275, *df* = 60, *p* = .026), with those married worrying less than singles, but no significant difference for married versus single regarding being anxious (*M* = 2.48 versus 2.10; *t* = 1.732, *df* = 60, *p* = .088). Finally, one-way ANOVAs were performed to determine if anxious and worrying differed based on year born (generational status) and all marital status categories. There were no significant differences.

## Discussion

This study provides important initial real time information regarding how African American Christians are feeling and coping with a critical crisis in our nation. Knowing, believing, and utilizing Scriptures seem to be essential elements of coping and protection against anxiety and worrying. Some of the most frequently mentioned chapters and passages contain powerful descriptors of God’s characteristics and/or have comforting words that can lead one to rely on and see God as a buffer, protector, and comforter (see Table 5).

**Table 5.** Selected Scriptures and Potential Coping/Buffering/Protective Descriptors (New International Version).

Scripture	Descriptors of God’s characteristics and/or comforting words
Psalm 91	Shelter; refuge; fortress; save from fowler’s snare and deadly pestilence; wings; dwelling; guard; rescue; loves me; rescues; protect.
Psalm 23	Shepherd; makes me to lie down; leads me; guides me; with me; rod and staff; comfort me; prepares a table; anoint my head; goodness and love follow me; will dwell in the house of the LORD forever.
2 Chronicles 7:14	My people; called by my name; hear from heaven; forgive their sins; heal their land.
Philippians 4:6–9	Rejoice in the Lord; do not be anxious about anything, but in every situation, by prayer and petition, with thanksgiving present your requests to God; the peace of God… will guard your hearts and minds in Christ Jesus.
Matthew 6:33	But seek first his kingdom and his righteousness and all these things will be given to you as well.
Joshua 1:9	Be strong and courageous; do not be afraid; do not be discouraged, for the Lord your God will be with you wherever you go.
Proverbs 3:5–6	Trust in the Lord with all your heart and lean not on your own understanding; in all your ways submit to him, and he will make your paths straight.

Many participants report that their prayer life increased and, like Scriptural passages, is serving a buffering and coping function. Pan et al. (2012) indicate the importance of Scripture and prayer in times of stress. This is consistent with existing literature which shows that prayer is a valuable coping mechanism and the most frequently employed of religious behaviors (Hood et al., 2018; Spilka & Ladd, 2013; Taylor et al., 2004). As noted by Entwistle et al. (2018), there are narratives in Scriptures that can be helpful during disasters and sufferings. While some respondents mention disaster narratives in the Bible, they mainly rely on specific passages.

Scriptures and prayer seem to be serving as buffers and protection from worrying and being anxious. Attending church services at least weekly prior to the pandemic is associated with being less worried and less anxious. Less worrying and being less anxious for those who attended church services more frequently, suggest not only possible buffering and coping effects, but also the importance of the social support function that churches and religious settings can provide (Merino, 2014; Taylor et al., 2004). Further, those married compared to singles who attended church services more frequently report less worrying. However, there is no significant difference between these group for being anxious.

Several respondents hesitated or raised concerns about the use of the words “religion” and “spirituality” as descriptors on the Likert-type scales. These words were perceived as too vague or not descriptive of them. One person refused to give an answer to these questions.

Unsolicited responses to these words were:

I like, I believe in Christ (15); I am a son of God (#16); I have a relationship with … (# 20, #21); With religion you can believe in the universe (#27); One can be religious and not a Christian (#29); Oh, I don’t like the word religious, I would say relationship (#31); These are tricky (#34); When I hear religious, it is like the Pharisees and Sadducees (#37); Ah, religion can mean many things. I go with the word Christian (#41); More of a relationship (#42); That is hard to say #44); Are we talking spiritual in the religious sense or in the yoga sense (#45); Its more than religion, it is born again, or faith based (#49); Religion is a set thing (#56); Relation with God, religion is man’s attempt to reach God (#65); and That’s kind of tricky (#72).

These unsolicited responses suggest that researchers, as well as those counseling and providing pastoral care with African American Christians, need to use appropriate descriptors in their communication. Thus, there is a need for clarity and greater understanding regarding the best terminology that resonates with this population, as well as what these terms mean to them (Mattis, 2000).

## Implications of COVID-19 for Counseling, Ministry, and Pastoral Care

Since respondents used social media at a high-rate, churches need to continue to incorporate ways to use such media in providing spiritual guidance, fellowship, and comfort and to encourage and prepare members to do so. Second, churches are encouraged to proactively prepare themselves and members for potential future crises like COVID-19 when governmental shut down and isolation may reoccur. Third, when the COVID-19 pandemic is brought under control and regular services are resumed, an assessment should be made of the lessons learned from the crisis. Fourth, churches, pastoral care professionals, and counselors could be more intentional in helping individuals to use Psalms during times of crises, since they are the most frequently mentioned of the Scriptures that are helpful. Finally, attention to specific populations is still needed because African Americans, as well as other populations, often have specific needs that must be addressed (see for example, Armstrong, 2016; Brown & McCreary, 2014; Jackson, 2015). Without this specificity, one runs the risk of not attending to nuances that are often inherent in specific communities.

## Limitations and Areas for Future Research

A limitation of the study is the lack of random sampling, particularly for the quantitative portion. Thus, the sample does not represent the full range of African American Christians, since participants are mainly from one state and majority midwestern. Typically, one finds differences by age and sex on many variables in African American populations. The lack of such a finding in this study may be due to the method of sampling and the small sample sizes for these groups. Further, several of the participants knew the interviewer, thus possibly producing social desirability effects. Despite these limitations, this study provides valuable information on the importance of religion, spirituality, and religious institutions as means of coping and protection for African American individuals who have a strong belief in God during COVID-19. Such information is helpful for workers both in the Christian community, the counseling arena, pastoral care, and the broader fields of mental health.

Future studies are needed that involve a random and larger sample. The use of appropriate terminology and its meaning needs to be further explored. Studies of how other faiths and racial/ethnic groups are coping with the pandemic would be enlightening. While it is informative to see the initial impacts of the coronavirus pandemic on the lives of these participants in real time, there is also a need to study the continuing and long-term impact of crises like COVID-19 (Aten et al., 2019), for there is some evidence that suggests that impacts on behavior during crises are short-lived (Uecker, 2008).
